# Subbarrel Patterns in Somatosensory Cortical Barrels Can Emerge from Local Dynamic Instabilities

**DOI:** 10.1371/journal.pcbi.1000537

**Published:** 2009-10-16

**Authors:** Bard Ermentrout, Daniel J. Simons, Peter W. Land

**Affiliations:** 1 Department of Mathematics, University of Pittsburgh, Pittsburgh, Pennsylvannia, United States of America; 2 Department of Neurobiology, University of Pittsburgh, Pittsburgh, Pennsylvannia, United States of America; University College London, United Kingdom

## Abstract

Complex spatial patterning, common in the brain as well as in other biological systems, can emerge as a result of dynamic interactions that occur locally within developing structures. In the rodent somatosensory cortex, groups of neurons called “barrels” correspond to individual whiskers on the contralateral face. Barrels themselves often contain subbarrels organized into one of a few characteristic patterns. Here we demonstrate that similar patterns can be simulated by means of local growth-promoting and growth-retarding interactions within the circular domains of single barrels. The model correctly predicts that larger barrels contain more spatially complex subbarrel patterns, suggesting that the development of barrels and of the patterns within them may be understood in terms of some relatively simple dynamic processes. We also simulate the full nonlinear equations to demonstrate the predictive value of our linear analysis. Finally, we show that the pattern formation is robust with respect to the geometry of the barrel by simulating patterns on a realistically shaped barrel domain. This work shows how simple pattern forming mechanisms can explain neural wiring both qualitatively and quantitatively even in complex and irregular domains.

## Author Summary

Complex spatial patterning, common in the brain as well as in other biological systems, can emerge as a result of dynamic interactions that occur locally within developing structures. In rodent somatosensory cortex, groups of neurons called “barrels” correspond to individual whiskers on the contralateral face. Barrels themselves often contain subbarrels organized into one of a few characteristic patterns. We suggest that these so-called subbarrel patterns arise spontaneously during development through a pattern-forming instability. We use a simple chemotaxis and branching model to explain the patterns and their dependence on the size of the barrel.

## Introduction

Mechanisms underlying the attainment of the central nervous system's highly structured organization are varied and numerous. The identification of developmentally regulated molecular signals are critically important for understanding neural function as well as fundamental processes of disease and repair. The complexity of the details notwithstanding, it is likely that many aspects of neural development can be understood in terms of relatively simple operational principles that govern the specific interactions among neurons and/or other elements, e.g., glia. Spatial patterns such as coat markings in animal skin and colors and textures in seashells are ubiquitous in biology, and theoretical studies have been able to account for a remarkable variety of them using models based on dynamical interactions among surprisingly small numbers of factors [1]–[4]. In many cases, quite complex patterns can emerge as a result of facilitatory – or positive – interactions among near-neighbor elements and converse suppressive –, or negative –, interactions more distally. This pattern of interaction has a long history in sensory neuroscience starting with the classic work by Hartline and Ratliff on lateral inhibition in the limulus retina [5] and its extension to models of visual cortex development [6].

The rodent somatosensory cortex contains striking spatial patterns of neuronal cell bodies and processes wherein discrete anatomical structures in layer IV called “barrels” correspond functionally with the representation of well-defined body surfaces [7],[8]. In the face area individual barrels, which are somewhat circular in shape, are related one-to-one to individual whiskers. The overall pattern of barrels is isomorphic with the pattern of mystacial vibrissae, reflecting a strong influence of afferent fiber systems in establishing the pattern (see Discussion). Viewed with cytochrome oxidase staining, individual barrels themselves appear heterogeneous, with regions of intense staining separated by narrow, often sinuous zones of less dense reactivity [9]. The cytochrome dense regions form “subbarrel” domains that correspond to cyto- and myeloarchitecture and that are enriched with thalamocortical axon terminals [10],[11]. Interestingly, subbarrels comprise a limited number of spatial patterns, with certain patterns more likely to appear in barrels corresponding to some whiskers than in others. The observed patterns are highly reminiscent of canonical patterns that populate circular domains containing diffusible media [1]. Here, we use a relatively simple model of chemoattraction and diffusion to simulate subbarrel patterning, and we test the model's predictions about the effect of barrel size on the resulting patterns. We find that predictable and sometimes quite complex subbarrel patterns can emerge as a result of interactions occurring locally and dynamically within the circular domain of the barrel.

## Results

**Figure 1:**
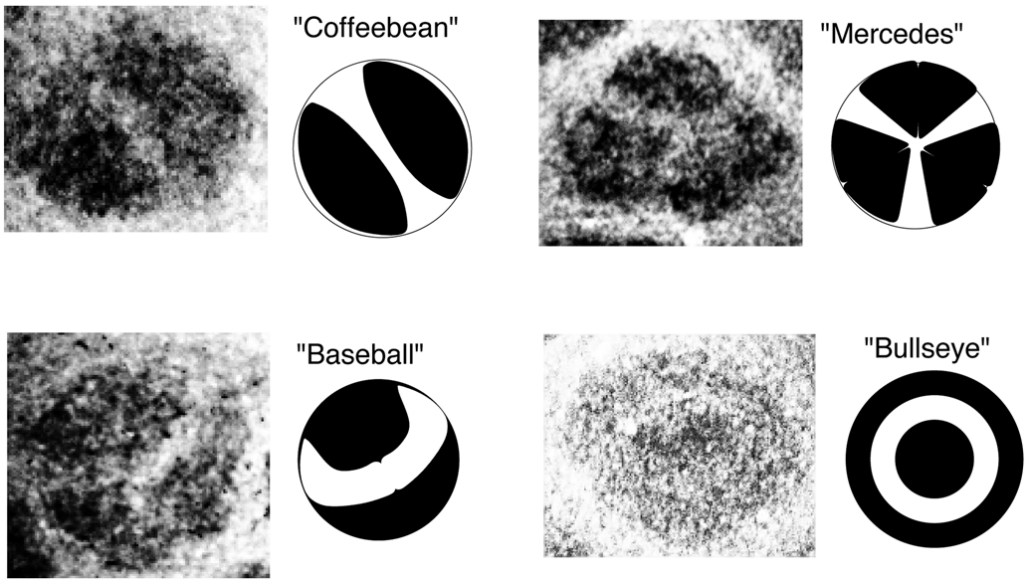
Examples of individual cytochrome oxidase stained barrels showing the four basic subbarrel patterns, illustrated schematically to the right of the corresponding photomicrograph. See also Fig. 4 [10]. Each sub-barrel is approximately 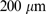 in diameter (see text and figure 3).

Cytochrome oxidase staining of individual whisker barrels reveals that there are patterns in the innervation of thalamic axons and that these patterns belong to only a few different classes. Figure 1 shows an example of each of the subbarrel types classified by [10] accompanied by an abstract representation of the pattern. [10] denoted the patterns as as coffee bean (cb), baseball (bb), bull's-eye (be) and mercedes (me). Smaller barrels and those found in the mouse primary somatosensory cortex have either no discernible patterns. The limited variety of patterns observed suggests that these are not random but rather are a consequence of some self-organizing principle such as seen in many other pattern forming systems. Indeed, the sub-barrels strongly resemble patterns seen on a vibrating circular drum.

There are many plausible models for pattern formation during neural development. By way of illustration and to show the underlying concepts, we will use a variant of the Keller-Segel [12] equations for chemotaxis. In our formulation, we suggest that thalamocortical axons within a single barrel undergo growth, pruning, random motion, and chemoattraction. We suppose that the axons or perhaps their target cells produce a chemical which diffuses, degrades and attracts other axons. We introduce 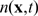 which represents the density of thalamocortical axons and 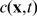 representing the concentration of the chemoattractant. 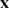 is the spatial position in the barrel which we take to be a disk of radius 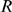. The equations we analyze have the following form:
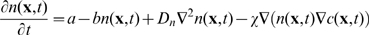
(1)

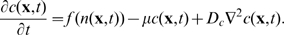
(2)The parameter 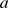 represents the production of new axon branches, 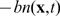 is the pruning, 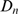 is the diffusion of the axons, and 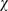 is degree of attraction of the chemoattractant 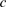. Henceforth, we assume that 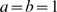 so that in absence of any interactions, the axons uniformly fill the barrel with a density of 1. The function 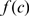 is monotone increasing and represents the production of 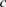 from the axons with density 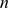. In simulations and analysis, we choose it to be 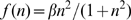 which saturates to 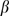 as 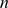 increases. The term 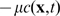 is the decay of the chemoattractant and 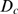 is its diffusion in the barrel. In absence of any spatial interactions there is a homogeneous equilibrium solution, 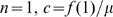. As this is a partial differential equation on the disk, we must specify boundary conditions. We choose “no flux” conditions, that is, there is no movement of axons or chemoattractant out of the barrel. Another possible choice which we discuss later in this paper is to set the value at the boundary to be the spatially homogeneous equilibrium state, 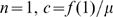.

### Basic ideas of pattern formation

**Figure 2:**
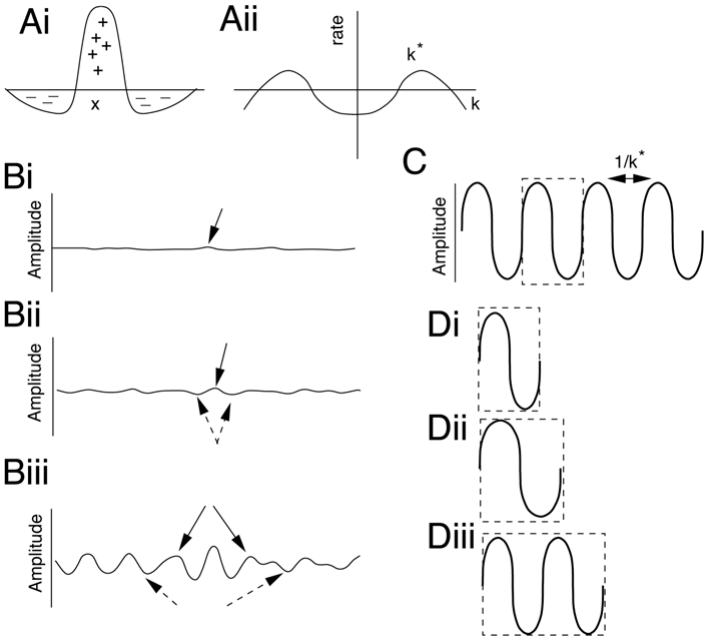
Basics of pattern formation in one dimension. A) Spatial interactions of the “surround inhibition”- or “Mexican hat” type (Ai) and its Fourier transform (Aii); note peaks at nonzero values of k. B) Interactions destabilize the uniform state. (Bi) small inhomogeneities (solid arrow) are amplified (Bii) by local positive feedback (solid arrow) while neighboring regions are depressed (dashed arrows). In Biii, because of the depression, neighboring regions are amplified (solid arrows) and their outer neighbors are in turn depressed (dashed arrows). C) Final patterned state. D) The complexity of the pattern is determined by the size of the domain. Di) there is a minimal length scale for creating a pattern; Dii) as domain size increases, the pattern expands to fill it; Diii) if the domain is large enough, a repeat of the pattern is inserted.

The main idea of spontaneous pattern formation is to show that spatially homogeneous activity in a model is unstable to perturbations that have a characteristic wave-length but stable to other perturbations. Thus, those in the unstable regime will grow and produce a *symmetry-breaking instability*. The classic way to implement this type of instability in biological systems is to have *lateral inhibition* in the model. Equations (1–2) have lateral inhibition “hidden” within their structure as we will see. Imagine a small heterogeneity in the density of thalamocortical axons, 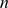 at some spatial region (figure 2Bi). This induces an increase in the chemoattractant, 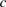 which draws neighboring axons up the chemical gradient. The result is fewer axons in the immediate neighborhood around this point (dashed arrows in figure 2Bii). Less chemoattractant is produced since there are fewer axons. This leads to local minima in 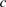 and thus axons move away forming secondary peaks (solid arrows, figure 2Biii) which in turn produce secondary valleys (dashed arrows.) The net result of these lateral interactions is a periodic pattern in one-spatial dimension (Fig. 2C). The spatial scale of the pattern is dependent on the diffusion of the chemoattractant, the spread of the axons, and the degree of chemotaxis. That is, in Figure 2C the distance between peaks is completely determined by these physical parameters. In small domains there may be only a single peak (or even no pattern) while in larger domains there may be many (Figs. 2Di–Dii). In this sense, the larger domains have more complicated patterns. The granularity of the pattern is determined by physical and chemical properties of the elements, so that the larger the domain, the more peaks and valleys possible. (We will discuss this sequence of figures further in the linear stability analysis.) Here, we have described a one-dimensional pattern. In the barrels, the radius of the barrel plays the role of domain length, so that larger barrels should have more peaks and thus more complex two-dimensional patterns, corresponding to subbarrels. In the next section, we make these intuitive arguments mathematically precise by analyzing equations (1–2).

### Linear stability theory

We assume that in equation (1), 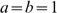 and in equation (2) that 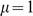 We assume there are no-flux boundary conditions. This means that 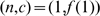 is a spatially constant steady state solution. We linearize the model equations about the equilibrium, 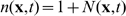 and 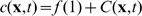 where 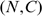 are small perturbations. To linear order,
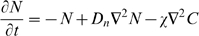


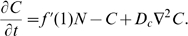
We note that the only spatial operator in the linearized equations is the Laplacian. Let 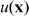 be an eigenfunction of the Laplacian on the barrel domain with no-flux boundary conditions with eigenvalue 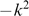:
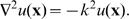
Then the general solution to the linear equations is
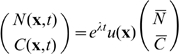
where 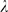 is an eigenvalue and 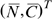 is the corresponding constant eigenvector for the matrix:
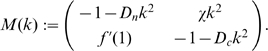
(3)If there are any values of 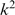 such that the real part of the eigenvalue, 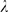, is positive, then the homogeneous equilibrium will be unstable, and we can expect patterns to grow that have the basic shape of the eigenfunction, 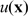. All parameters in the matrix are positive including 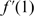. For two-dimensional matrices, a necessary and sufficient condition for eigenvalues having negative real parts is that the trace (sum of diagonals) be negative and the determinant be positive. Clearly the trace is negative for all 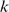 and the determinant is
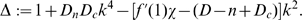
(4)For both small and large values of 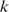, the determinant is positive. If 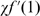 is smaller than 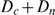, then the determinant is positive for all 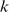 and there can be no pattern forming instability, since the homogeneous state is always stable. However, if the chemotaxis is large enough and the production term, 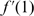, is large, then the term in the square brackets can be positive, and it is thus possible for the determinant to be negative. Thus, we want to find the value of 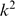 which minimizes the determinant and we want this minimum to be negative. The minimum occurs when
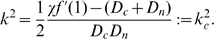
There are several parameters we could vary to produce an instability. For reasons of convenience, we use the diffusion of the chemoattractant as our main parameter and find that the determinant vanishes when
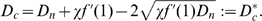
That is, if 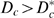 then the spatially uniform state is stable and if it is smaller than 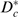, the spatially uniform state is unstable. To simplify this even further and for use when we simulate the full nonlinear system, we set 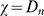, so that
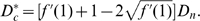
(5)As long as 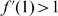, we can find a positive value of 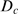 which produces the pattern forming instability. With 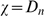 and 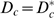, the critical value of 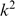 is
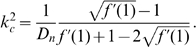
(6)The larger the value of 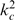, the finer will be the pattern that arises from the loss of stability of the uniform state. That is, for small values of 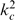 the pattern will have little spatial variation and would, e.g., correspond to a mouse barrel. Small values of 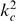 are associated with large values of 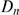; the larger is 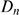, the coarser will be the pattern. We finally note that 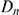 effectively sets the size of our system: large values of 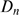 correspond to small domains and small values of 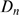 correspond to large domains.


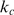
 is set by the physiology, so that we can regard the “size” of the barrel to be the “bifurcation parameter.” Since the domain size is finite, the set of values that 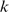 can take is discrete. Thus, as the characteristic length of the domain increases, there will be jumps in the number of local extrema in the patterns. This is why the pattern appears to stretch in the transition shown in figure 2D1–3.

### The form of the patterns

So far, the description of instability has been general in that we have not made use of the shape or size of the domain (the barrels). In this section, we state our main results which describe the patterns one expects to form spontaneously as we decrease the diffusivity of the chemoattractant. Recall from the previous section, that the spatial form of the patterns is determined by 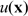, the eigenfunction for the Laplacian. In this section we consider a simple disk-shaped region, because the solutions are explicit. Later, we numerically compute eigenfunctions for an irregular domain and see qualitatively similar results.

For a disk-shaped domain, it is convenient to write the eigenvalue problem in polar coordinates, 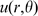 so that we must solve:
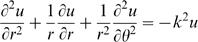
subject to no flux boundary conditions: 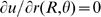. Since 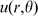 must be 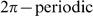 in 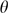, we write 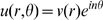 where 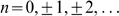 and 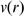 satisfies the ordinary differential equation
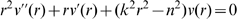
(7)and 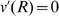 (Here 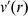 denotes the derivative of 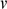 with respect to 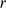.) Equation (7) is Bessel's differential equation and has solutions that are well-defined at 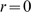,
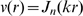
where 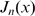 is the Bessel function of the first kind of order 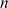 We need 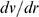 to vanish at 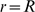 the radius of the barrel. Thus, 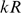 must be a zero of the derivative of 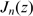. If we had chosen a different boundary condition (such as the chemoattractant concentration is fixed at the edge of the barrel), then instead of 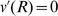, we would have 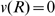 and this would imply that 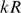 must be a zero of 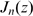 rather than a zero of its derivative.

**Figure 3:**
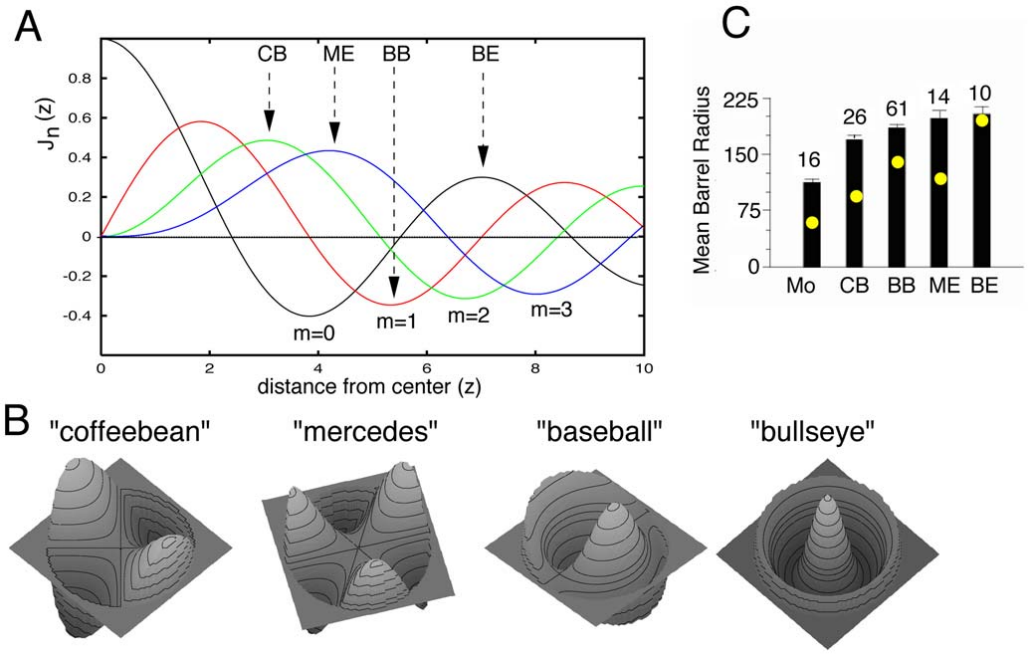
Patterns on a disk. A) Plots of the first 4 (denoted by 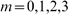) Bessel functions, 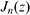 as a function of distance (z) from disk center. Arrowheads delineate locations of 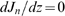, corresponding to the minimal disk size where a given pattern will first emerge. B) Three dimensional views of the eigenfunctions showing their correspondence to the four basic subbarrel patterns. C) Mean radius of barrels containing particular subbarrel patterns (Mo(use)  =  no pattern). Numbers above each bar indicate number of barrels measured. Dots within each bar indicate the theoretical size of the barrel expected to contain that subbarrel pattern.

Figure 3A shows the first 4 Bessel functions, orders 0–3, plotted with respect to distance from the center of the barrel. When the conditions at the boundary of the barrel are no flux, 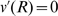, then we are interested in the values of 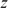 such that 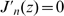, that is, the maxima and minima of the Bessel functions. If, instead, we use fixed boundary conditions, then we are interested in the zero crossings of the Bessel functions. Since we use no-flux conditions, the critical value, 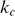 and the radius of the barrel, 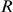 must be such that 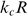 is one of the maxima or minima of the curves in this figure. Recall that 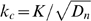 from equation (6) and 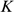 is determined solely by the function 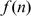. Thus, we want 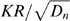 to be an extreme value of one of the Bessel functions. By varying the radius 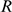 (or equivalently, the diffusion, 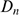, which is convenient for numerical purposes), we can fix the pattern that will arise as we lose stability of the homogeneous state. We can use figure 3A to determine the shape of the emergent pattern. Fix 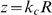 to lie on one of the maxima or minima of the plotted curves. The order of the curve, 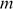, indicates the number of maxima/minima we encounter as we move circumferentially around the perimeter of the barrel. The number of maxima/minima of the curve between 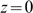 and 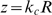 indicates how many maxima/minima will be encountered as we move radially from the center of the barrel to its edge. The two simplest examples to understand are the mercedes and the bullseye. Since the order is zero for the bullseye, the pattern is rotationally symmetric. As we move from the center outward, there will be a peak at the center, followed by a valley, terminating with a peak at the edge. By contrast, the mercedes is order 3. At the edge the density of axons will show three maxima and three minima while at the center the density is at the homogeneous state. Consider the coffeebean. The density shows two maxima and two minima as we traverse the circumference of the barrel with the center showing the background density. The order of the baseball is 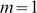, so on the perimeter there will be one maximum and one minimum. However, as we move from the perimeter inward, we will encounter a maximum between the perimeter and the center. We remark that since the theory outlined here is linear, changing the sign of the curves in figure 3A yields more patterns which are not qualitatively different. However, consider the 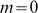 curve and the point at the first minimum (around 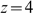). As plotted this curve yields a pattern that is rotationally symmetric with a maximum density at the center and a minimum density at the edge. Reversing the sign of 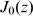, we obtain a pattern with a minimum in the center and a maximum at the perimeter, a ring-like pattern. Changing the sign for patterns with 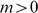 is equivalent to rotating the pattern. If 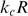 is smaller than the first extreme value of any of the curves (approximately 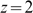 on the 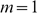 curve), then there can be *no* patterns; this would be the case for the mouse barrels or smaller barrels in the rat.

The patterns in Figure 3B are universal in that they emerge with any dynamic pattern-forming mechanism; other reaction-diffusion models or even a Hebbian learning model would produce the same patterns at least to linear order. The stereotypy of patterns is a consequence of the rotational symmetry of the problem; in fact, symmetric bifurcation theory allows us to conclude that the *nonlinear* patterns that emerge from the homogeneous state are also universal [13]. Aside from the shapes of the patterns, how can we test the idea that spontaneous pattern formation underlies the subbarrel structures? As noted above, the size of the domain is a key determinant. In Figure 3A, the zeros of the derivatives of the Bessel functions corresponding to our four pattern classes determine the minimal barrel sizes needed for the patterns, and thus we can deduce a size principle from them. The coffeebean pattern is the simplest and should occur in the smaller barrels, whereas the bullseye is the most complex and should occur only in the largest barrels. The mercedes and the baseball will be in intermediately sized barrels. Finally, very small barrels should have minimal structure and an almost uniform pattern, i.e., no sub-barrels. From Figure 3A, we make the following size prediction: 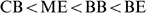. The areas of 113 barrels were measured (see Methods). We find that barrels containing a bullseye pattern are the largest (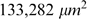), and barrels containing coffeebeans are smallest (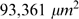); those containing mercedes (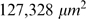) and baseballs (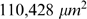) are intermediate in area. The theory reverses the baseballs and the mercedes patterns. We note that the mercedes pattern is distinctive and easy to identify, whereas the baseball pattern can be confused with the coffeebean, because both have two main lobes, the baseball being slightly curved. Thus, it is possible that some coffeebeans were misclassified as baseballs, and this would tend to lower the mean area for baseball-containing barrels. Moreover, baseball-containing and mercedes-containing barrels are virtually equivalent in size. In Figure 3C, areal measurements are transformed to estimates of radius and plotted with respect to values predicted from the model; values have been scaled so that the largest simulated barrel (BE) has a radius of 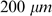, equivalent to the average value for real barrels containing the BE pattern. Note that, as predicted, patterns are not observed in mouse barrels (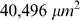) nor are they evident in similarly small barrels in rats corresponding to the small peri-oral sinus hairs (Land and Erickson, 2005). Regression analysis of the five pairs of real and theoretical radius means indicated good, trend-level correspondence (p = 0.08); results were more robust when values for BB and ME were reversed in order (p = .05).

**Figure 4:**
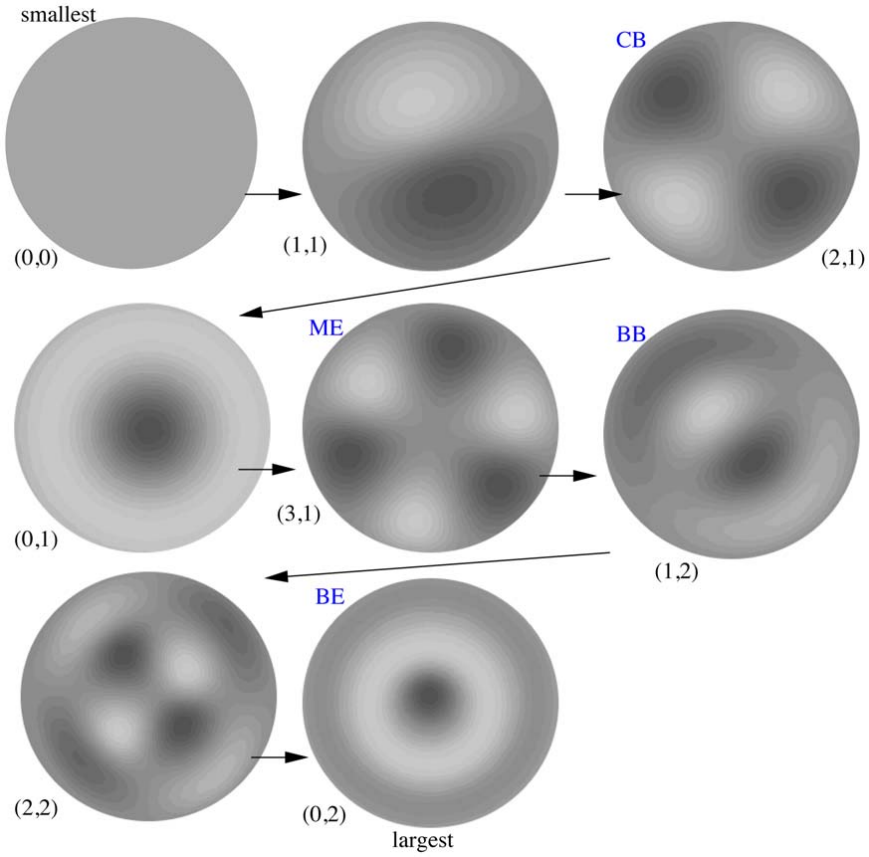
Sequence of possible patterns assuming no-flux boundary conditions and size of the barrel as a parameter. Dark regions correspond to highest density of thalamocortical axons. White areas correspond to density less than background. Blue labels are the named patterns seen in the data. All barrels are drawn at the same diameter. Numbers in parentheses, 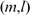, denote the order of the Bessel function, 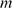, and its zero, 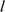; see figure 3A.

Figure 4 shows a complete sequence of patterns formed as the radius increases from small to large values. Beneath each figure, we show an ordered pair 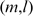 corresponding to the Bessel function of order 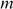 and the 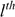 zero of the derivative. We have also labeled the patterns corresponding to the Land and Erickson classification. Pattern (0,0) would correspond to a mouse barrel. The pattern (1,1) would likely be degenerate case; it would appear as a half-barrel inasmuch as the other half is devoid of axon terminals or perhaps as an unpatterned barrel with a small local region of axon terminals. There are other patterns that we have not found in the sub-barrel structures, for example, the (2,2) pattern is rather striking. There is no reason why this pattern should not appear as a sub-barrel pattern; so far, we have not found an example in our database of images.

Throughout this discussion, we used no-flux boundary conditions to obtain the patterns. A similar sequence occurs with fixed boundary conditions. In fact, it follows from the general theory of second order linear differential equations [14], chapter 9, that there will be a sequence of solutions that have an increasing number of extrema as the domain size increases. Thus, there is nothing special about our choice of conditions at the edge of the barrel.

### Numerical simulations

**Figure 5:**
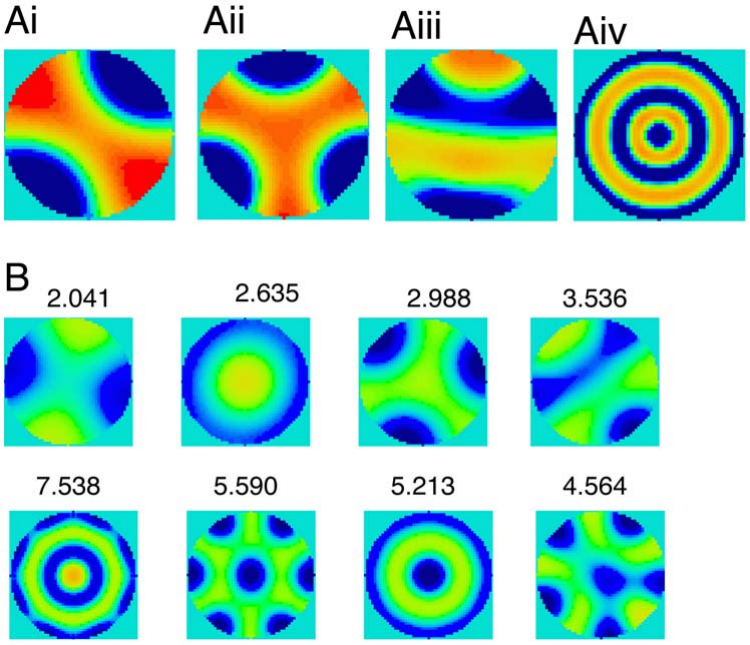
Numerical solutions to the full nonlinear equations. 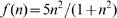
 and 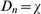. A) Numerically computed representations of the 4 standard patterns. Ai) 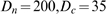; Aii) 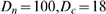; Aiii) 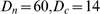; A4) 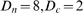. B) Sequence of patterns with 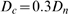 as 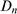 decreases. Numbers next to patterns are 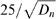 and correspond to a dimensionless size. (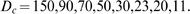).

The above analysis suggests the types of patterns that are possible for the full non-linear system for parameters near the loss of stability of the constant state. In this section, we numerically solve equations (1) and (2) on a fixed radius disk and vary the values of chemotaxis and diffusion. Figure 5A shows representative solutions to the full nonlinear problem when the initial data is chosen to be a small random perturbation around the homogeneous steady state. Clearly, the nonlinear patterns are quite similar to those predicted from the linear analysis. Figure 5B fixes the ratio 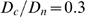 and varies 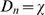 from a large value (corresponding to the smallest barrels) to a small value (corresponding to the largest barrels). Numbers next to the patterns indicate a relative size of the barrel. The resulting nonlinear patterns include all four of the reported classes of patterns including at least two patterns that could be considered bullseyes (labeled 5.213 and 7.538). The mercedes (2.988), and baseball (3.536) patterns are adjacent which is consistent with the linear prediction shown in figure 3C. The mercedes has three-fold symmetry and for larger domains, the model shows patterns that have five- and six-fold symmetry (4.564 and 5.590 respectively). These latter patterns were not depicted in the linear analysis as they correspond to Bessel functions of order 5 and 6 respectively. Figures 5A,B thus show that the nonlinear solutions are consistent with an ordering of coffeebean smallest and bullseye largest, with mercedes and baseball in-between.

### Realistic barrel shapes

**Figure 6:**
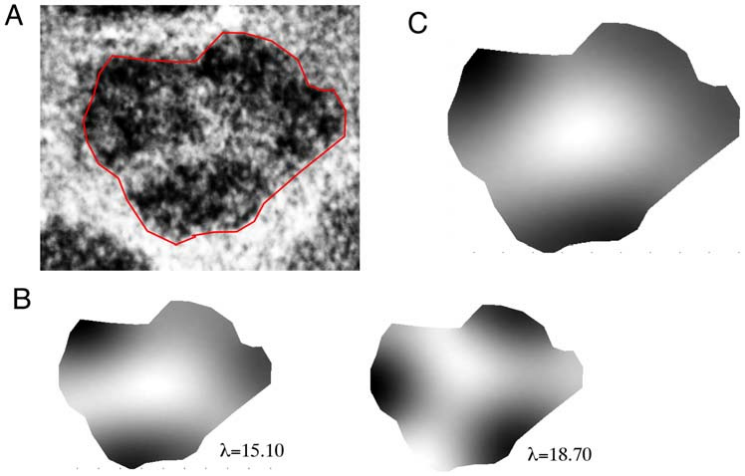
Irregular barrel with a mercedes pattern. (A) Image of the actual barrel showing the piecewise linear approximation of the boundary (red). (B) Two eigenfunctions with nearby eigenvalues. (C) Superposition of two patterns in (B).

For mathematical simplicity, we have treated the barrels as disks, but real barrels have less regular shapes. A natural question is whether the qualitative shapes of the patterns are robust to irregularities in the actual barrel domains. In order to examine this, we chose a specific barrel with a very clear mercedes pattern (see figure 6A) and traced its perimeter as a series of line segments. We exported the coordinates of the perimeter to MATLAB and used the PDEToolBox to numerically compute the eigenvalues and eigenfunctions of the Laplacian on this irregular domain. Figure 6B shows two eigenfunctions along with their corresponding eigenvalues chosen to have the structure of a mercedes pattern. The disk has rotational symmetry, so that the two corresponding eigenfunctions are just rotations of each other and have identical eigenvalues. In the irregular domain shown here, the “rotated” pattern has a slightly different eigenvalue. Nevertheless, the two eigenvalues are quite close, so we expect that the patterns that arise will be a combination of the two patterns. Indeed, when we we add the two eigenfunctions together we get the full pattern shown in figure 6C which matches the experimental pattern quite well.

## Discussion

Here we demonstrate that appropriate and complex anatomical patterns can be understood in the context of general pattern forming mechanisms in a circular domain. Emergence of spatial patterning is common in development, and a number of such processes have been modeled as dynamical systems. In a classic paper on morphogenesis, Turing [15] showed that diffusive interactions between chemical reagents are sufficient to produce spatial patterns. Such models have been used to explain a host of biological patterns, including markings on seashells and animal skins [1],[4]. Patterns similar to ocular dominance stripes in visual cortex can emerge from a relatively homogeneous substrate when its spatially unstructured state is induced to become unstable [6],[16]. In such models, the key component required for pattern formation is a mechanism similar to *lateral inhibition*, that is, local interactions which facilitate growth/activity and distant interactions which suppress it [17]. Here, we produced sub-barrel patterns using a simple model for growth, pruning, and rearrangement of thalamocortical axons. These interactions were sufficient to generate the required lateral inhibitory interactions. As illustrated in Figure 2, such interactions can produce multiple structured regions whose number and shape are in turn determined by the geometry and size of the domain. Small domains, like mouse barrels, yield a single, nearly homogeneously organized structure whereas larger domains, similar to rat barrels corresponding to the most densely innervated and largest facial whiskers, can support complex patterns.

Figures 3A,4 and 5 suggest that there could be a number of other subbarrel patterns, inasmuch as the four patterns described here by no means exhaust all possibilities. For example, the model shows a pattern (0,2) in Figure 4 for the zero-order Bessel function in which there is a single dark center with a lighter surround. This and some other patterns predicted by the theory have not been observed in rat or mouse barrels; this may reflect additional, specific biological constraints not captured by our simple model. In this regard, it is interesting to speculate that more complex patterns, e.g., four- or five-lobed ones (e.g. in figure 5), might be visible in species such as the rabbit, capybara and brush-tailed possum that have barrels even larger than those in rats [7],[18].

There are many possible mechanisms for pattern formation. The present model is based on chemotaxis and diffusion, though other processes, employing chemorepulsion and/or additionally involving activity-dependent competition for resources, are also plausible. Here we use growing thalamocortical axons as the fundamental, interacting elements, as virtually all empirical studies support a central role of these afferent fibers in establishing the basic pattern of barrels within the face area of the primary somatosensory cortex (e.g.[19],[20]). Subbarrel patterns also appear to be organized with respect to growing thalamocortical axons, with the patterns developing gradually and becoming recognizable in the second week of postnatal life, after the initial in-growth of the axons and the emergence of the larger barrel structure [11]. During this time, thalamocortical axon arbors become more geometrically complex, progressing from a relatively simple and sparse branching to a dense mesh work of overlapping branches [21]. Thus, at the time sub-barrels begin to emerge, axon density may have attained a level that permits the types of near-distance interactions that form the basis of the model presented here.

The mechanism(s) by which thalamocortical axons interact with each other and with the cortical neurons themselves are largely unknown, and our model makes no explicit assumption – or prediction – regarding the detailed processes underlying subbarrel formation. Indeed, our model employs only two key variables – thalamocortical axon density and chemoattractant concentration, though numerous morphogenetic factors involving the growth and elaboration of axons, dendrites and synapses are almost certainly involved in establishing the organization of cell bodies and neuropil within each barrel. A number of molecules thought to be important for barrel formation are themselves regulated by neuronal activity, though at present the role of activity in the formation of barrels or subbarrels remains unclear [22]. In this regard, it is important to note that formation of barrels relies on whisker-specific cues, whereas subbarrel patterns must develop from cues common to the same whisker. Thus barrel and subbarrel development may depend on different mechanisms. It is nonetheless interesting that polygon-shaped structures remarkably similar in shape and overall spatial arrangement to barrels can be generated by competitive interactions among outwardly directed forces emanating from center points contained within neighboring Dirichlet domains [23]. Taken together with the present results, the findings suggest that, though the detailed biological mechanisms underlying barrel formation are likely to be varied and complex, the basic structure of the barrels and of the patterns within them may be understood in terms of some relatively simple dynamic processes.

One question we have not addressed in this paper is why there are sub-barrel structures at all. The *null hypothesis* is that they arise simply as a consequence of the mechanisms for axon targeting; that is, they are epiphenomena of the growth process. They may nonetheless provide a functional role. As the size of the barrel becomes larger, it may be necessary to develop multiple local circuits. [24] have found local angular tuning domains in rat barrels. Sub-barrels may facilitate the creation of these local circuits.

## Materials and Methods

For the biological portion of this study we reanalyzed 113 rat barrels whose subbarrel patterns were described previously (Land and Erickson, 2005). These specimens were derived from layer 4 of the somatosensory cortex in young rats ranging in age from postnatal day 10 (P-10) through P-16. Cortices were prepared as tangential, in vitro slices. Slices were prepared by standard methods. Live slices subsequently were fixed in 4% paraformaldehyde, sectioned at 80 µm and stained histochemically for cytochrome oxidase (CO) (Land and Simons, 1985). Each of the barrels chosen for the current analyses contained one of four basic subbarrel arrangements that are recognized based upon the pattern of CO-dark and CO-light zones. We acquired images of CO-stained barrels with a SPOT RT digital camera (Diagnostic Instruments, Sterling Heights, MI) using a Kodak 47B Wratten filter and imported them into Photoshop (Adobe Systems Incorporated, San Jose, CA). To further enhance the contrast between CO-dark and CO-light regions, the original RGB color images first were converted to grayscale. We then applied the Equalize command, which finds the brightest and darkest values in the composite image and remaps them so that the brightest value is depicted as white and the darkest value as black. Resulting equalized images were analyzed using Scion Image (Scion Corporation, Frederick, MD). We used the Freehand Selection tool to outline the perimeter of CO-stained barrels and then exported the area data into a spreadsheet (Excel, Microsoft Corporation, Redmond, WA) We organized the data into groups of barrels that exhibited a particular subbarrel pattern (i.e., cb, me, etc.) and determined the mean and standard deviation of barrel areas associated with each pattern.

The nonlinear partial differential equations models were solved on a 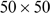 grid whose active elements were restricted to a circle of radius 25. For simplicity, we used Euler's method with a time step of 0.001. The eigenfunctions of the irregular domain were found using the PDE ToolBox from MATLAB (Protocol S1).

## Supporting Information

Protocol S1 Matlab code to obtain eigenfunctions for a realistic barrel shape.
(1 KB ZIP)
